# Using Facebook to Improve Participation in Colorectal Cancer Screening: Protocol for a Cluster Randomized Controlled Trial

**DOI:** 10.2196/86829

**Published:** 2026-05-14

**Authors:** Arlinda Ruco, Natalie Baker, Melissa Howse, Anne Sorvari, Jenna Jacobson, Diego Llovet, Jill Tinmouth, Rahim Moineddin, Nancy Noel Baxter

**Affiliations:** 1 Interdisciplinary Health Program St. Francis Xavier University Antigonish, NS Canada; 2 Li Ka Shing Knowledge Institute Unity Health Toronto Toronto, ON Canada; 3 Ted Rogers School of Management Toronto Metropolitan University Toronto, ON Canada; 4 Institute of Health Policy, Management and Evaluation University of Toronto Toronto, ON Canada; 5 Dalla Lana School of Public Health University of Toronto Toronto, ON Canada; 6 Faculty of Medicine and Health The University of Sydney New South Wales Australia

**Keywords:** cancer screening, mass screening, Facebook, social media, cluster randomized controlled trial, cluster RCT

## Abstract

**Background:**

Colorectal cancer (CRC) screening participation in Canada is lower than the national target, and interventions designed to increase screening participation are generally expensive and have limited impact. Social media can be used as an innovative strategy to increase participation in cancer screening, particularly Facebook (FB), as it is the most popular social media platform for the population eligible for CRC screening.

**Objective:**

The aim of this study is to report on the protocol for a pragmatic cluster randomized controlled trial that will test the effectiveness of CRC social media advertisements on user engagement and screening intention.

**Methods:**

The trial will target FB users aged 45 to 64 years who reside in the province of Ontario, Canada. There are 521 forward sortation areas (FSAs) in Ontario, and the randomization will be done at this level using the first 3 digits of the postal code. Rural and urban FSAs will be randomly allocated to one of the 6 study arms. In 4 arms, FB users will all receive one of 4 social media advertisements developed in previous studies, while in the fifth arm, a tailored strategy by sex will be tested. In the final arm, FB users will not be shown any advertisements. If users click on any of the advertisements, they will be directed to a webpage with more information on screening and a place to pledge their intention to screen for CRC. The study’s primary outcome will be tested as a count measure, defined as the number of people per FSA who pledge their intention to screen for CRC. User engagement metrics, including impressions, link clicks, cost per link click, link click-through rate, and user comments, will be tracked across the 5 trial arms with advertisements shown.

**Results:**

This cluster randomized controlled trial will provide evidence on the use of FB as a tool for delivering CRC screening messages and influencing screening intentions. The comparison of message types within a fixed campaign budget could identify which approaches promote user engagement in both urban and rural populations.

**Conclusions:**

This study has the potential to show that social media offers a cost-efficient, scalable approach to promote CRC screening. This approach is adaptable to other cancer screening programs and could provide evidence-based digital strategies for the promotion of cancer screening.

**Trial Registration:**

ClinicalTrials.gov NCT04296630; https://tinyurl.com/5y5k7yjb

**International Registered Report Identifier (IRRID):**

DERR1-10.2196/86829

## Introduction

### Background

Colorectal cancer (CRC) accounted for 10% of all new cancer cases in Canada; an estimated 25,200 patients were diagnosed, and of those, 9400 died from CRC in 2024 [1]. Screening has been found to reduce CRC mortality and is an important intervention to improve the burden of the disease [2]. Currently, persons eligible for screening in Ontario are mailed an invitation from the provincial screening program, ColonCancerCheck (CCC), requesting they contact their primary care provider to obtain a test kit [3], while persons without a primary care provider can call Health811 to determine their eligibility and order a Fecal Immunochemical Test kit [4]. However, despite the infrastructure and availability of organized programs, screening participation in Canada has plateaued at less than the 60% national target [3,5,6].

Interventions designed to increase screening participation are generally expensive, challenging to implement, and have limited impact [7-10]. For example, directly mailing test kits to participants is an effective intervention to increase participation [9]. Even with a conservative estimate of the kit cost at CAD $15 (US $11) [1] per kit, sending a Fecal Immunochemical Test kit to all individuals in Ontario eligible for screening—approximately 1.6 million people [11]—would result in a cost of more than CAD $24 million (US $17.6 million) for the CCC screening program, with a significant portion of these kits going unused. Other interventions, including patient navigation, can be extremely costly and challenging to implement as staff are required [7]. Incremental costs per person screened for patient navigation are estimated to range between CAD $541 (US $396.60) to CAD $812 (US $595.30) [12].

Social media has not been extensively explored in the context of improving participation in cancer screening [7-10], but the use of social media for health promotion and behavior change interventions has great promise [13-21]. A systematic review of social media interventions showed that this approach has been successfully used for lifestyle behavior change (physical activity and eating behaviors), weight reduction, improvements in quality of life, health knowledge, and self-efficacy [15]. Our systematic review and meta-analysis assessed the effectiveness of social media and mobile health (mHealth) interventions in promoting cancer screening (breast, cervical, CRC, lung, and prostate) [22]. Out of 39 included studies, 35 focused on mHealth and only 4 on social media [22]. Recently, Ontario Health (OH), a provincial agency responsible for the administration of the health care system, has tested the feasibility of using social media to deliver tailored breast cancer screening messages. The study was not designed to evaluate screening uptake, but the campaign was able to reach close to 60,000 women within 1 month (Karapetyan T, unpublished data, 2019). Social media has the potential to be an innovative strategy to increase participation in cancer screening, but it has not been adequately studied—this is a critical gap in knowledge.

Facebook (FB) is the most popular social media platform for people aged 50 to 74 years (the population eligible for CRC screening) [13,23]. Approximately 75% of those aged 55 years and older use FB, with approximately 80% of all FB users reporting daily use [13,23-25]. Therefore, most individuals of screen-eligible age have access to and use FB regularly, making it the ideal platform for this study.

### Objective and Aims

The objective of this study is to present the protocol for a pragmatic cluster randomized controlled trial (RCT) to test the effectiveness of social media advertisements for CRC screening on user engagement and screening intention. A cluster design was chosen based on the platform’s targeting options for advertisement campaigns. A future aim of the study will be to explore the effectiveness of the social media advertisements on actual screening uptake through the use of health administrative data.

## Methods

### Study Design

This will be a pragmatic cluster RCT testing social media advertisements for CRC screening through an advertisement campaign on FB (Meta Inc) in the province of Ontario, Canada, reported using the SPIRIT (Standard Protocol Items: Recommendations for Interventional Trials) checklist [26] and the CONSORT (Consolidated Standards of Reporting Trials) statement extension for cluster randomized trials [27].

**Figure 1:**
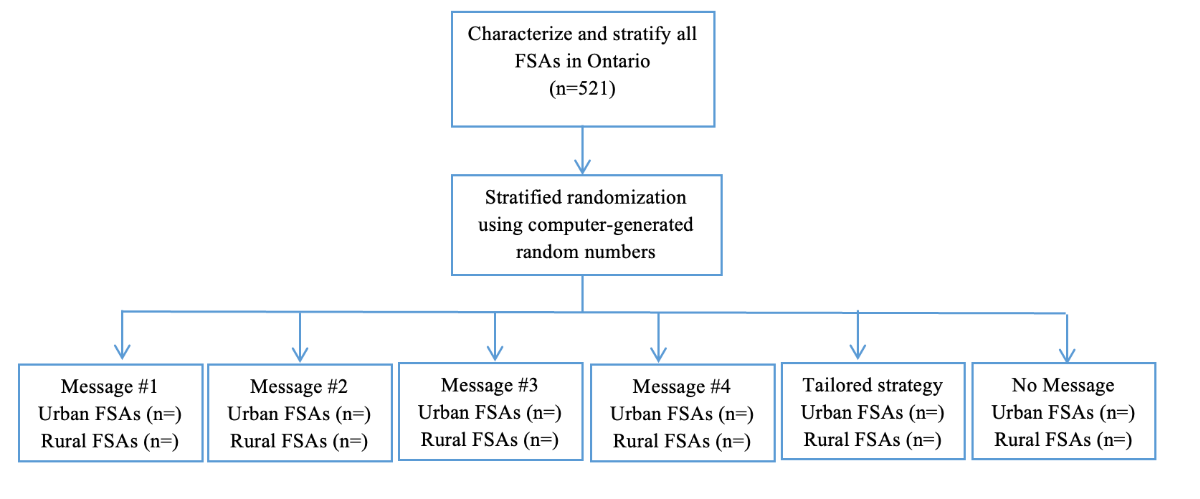
Study flow diagram. FSA: forward sortation area.

Advertisement campaigns on FB can be targeted based on demographics, including sex, age, and location of residence, determined by FB using regularly collected data from users [28]. The advertisement campaign manager can also determine the length of the campaign and the maximum budget based on daily spend or lifetime campaign budget. This cluster RCT will use Ontario’s forward sortation area (FSA), specifically the first 3 digits of the 6-digit postal code. FSAs are defined as urban or rural based on the second character of the FSA, where a 0 indicates a rural region, while all other digits refer to urban regions. On average, the population of each FSA in Ontario is 25,714 people, 30% of whom are estimated to be of screen-eligible age. Assuming at least 70% of screen-eligible individuals are FB users [25], we will have, on average, 5400 eligible individuals for each FSA. The FSAs will be assigned randomly to one of 6 trial arms (Figure 1) and all eligible FB users residing in the same FSA will be assigned to the same trial arm. The FB advertisement algorithm starts by filtering advertisements from their database based on the user’s profile and ranks them to identify the best candidates for the ad. The potential advertisements are further refined by a bidding process, where the advertisers bid on clicks and advertisements are ranked by the bid, the likelihood of engagement, and advertisement quality. The process is designed to ensure the most relevant advertisements are shown to users while optimizing advertiser budgets. FB uses machine learning to optimize the campaign in real time. If the advertisement is performing well, FB might automatically increase the delivery to reach more people. The advertisements in our campaign to promote CRC screening will be displayed to FB users aged 45 to 64 years (the most appropriate age range option available through FB Ads Manager) based on the FB advertisement algorithm.

Because randomization will occur at the FSA level, we will not collect identifying information on individual targeted FB users and will not link outcomes to individual users, and thus will be unable to determine outcomes at the individual level.

### Ethical Considerations

The Research Ethics Board of Unity Health Toronto, Toronto, Ontario, Canada, approved the protocol for this study (REB#20-005). Given the nature of this trial, informed consent from individual FB users is not possible and thus permission to conduct this study without consent from individual FB users was granted by the Research Ethics Board. All data that will be collected in this study will be in aggregate, summary form and include anonymized data only. No personal identifiable information will be collected and only the research team will have access to the collected data.

### Intervention and Comparison

Prior to the cluster RCT, we developed the social media advertisement (text and image) combinations to be used by conducting focus groups with individuals of screen-eligible age. Participants were presented with social media advertisements (both text and image) and were asked to provide feedback. Based on the feedback, recommendations were developed for potential use [29]. We then engaged a public relations firm to help refine the creative concepts for our target audience. Once we had the social media advertisements ready, we conducted 2 split (A and B) tests with a total of 6 creative concepts using FB. Split testing is a method that allows changes to a specific advertisement variable (image or text) and allows testing of 2 versions of the advertisement with different groups of FB users to see which advertisement is preferred by the target audience. We conducted these split tests in the province of British Columbia to prevent potential cluster RCT participants from seeing this test advertisement campaign. The top-performing creative advertisement concepts were then selected to be used in the cluster RCT (Multimedia Appendix 1).

**Figure 2:**
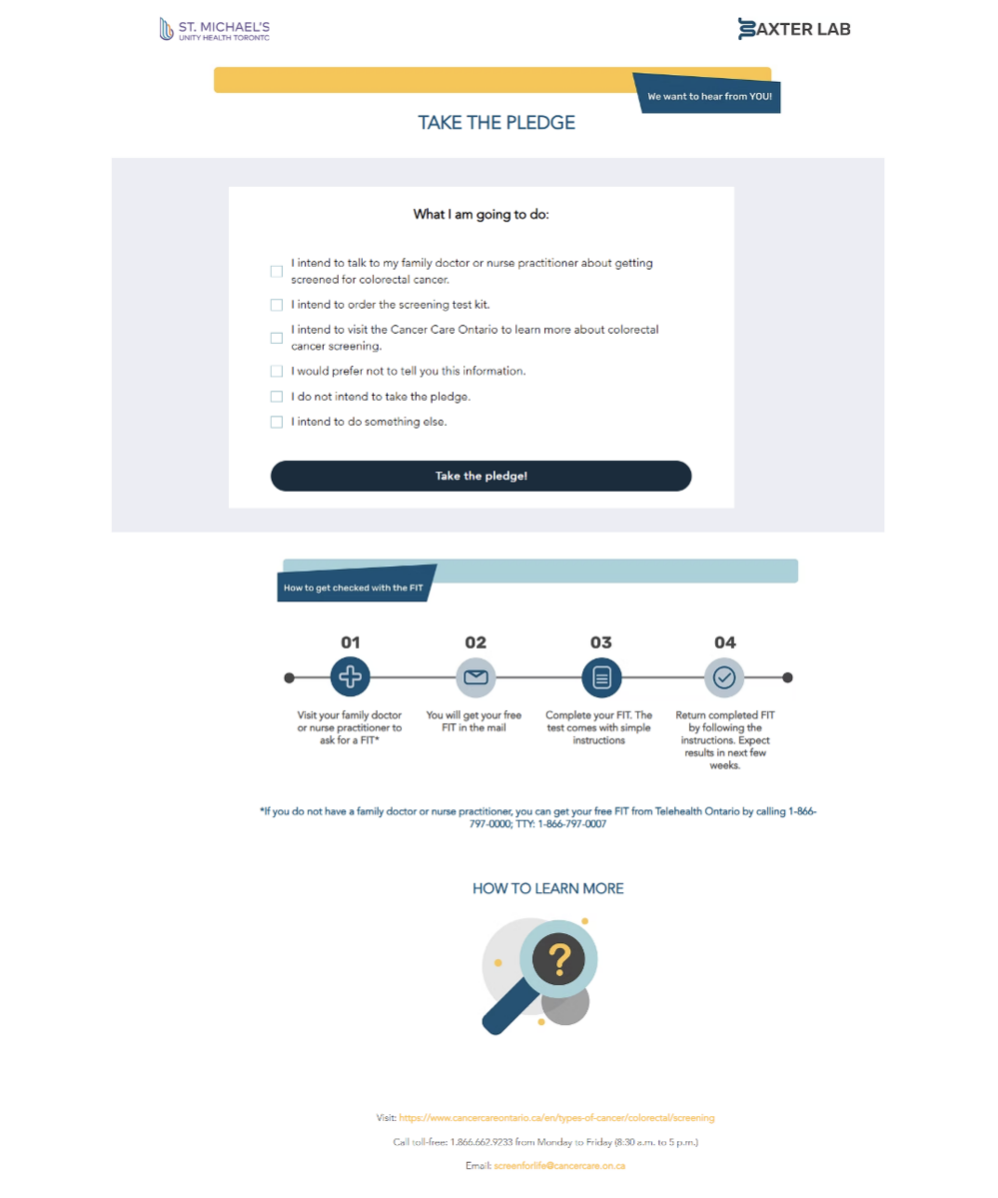
Screening intention outcome as will be captured on the study website.

If a user clicks on one of the FB advertisements (Multimedia Appendix 1), they will be redirected to a webpage with cancer screening information [30]. The landing page has content included in the CCC invitation letters [31] and information available on the OH website (as these have been extensively tested with the screen-eligible population, are concise, and provide relevant information in lay language). In addition, links to the OH website will be provided, including an email and telephone number for any additional queries (Figure 2).

The webpage prompts users to indicate their intention to undergo CRC screening (primary outcome) by completing a pledge (Figure 2). OH used a similar method in a recent study to assess screening intentions through targeted messaging for breast cancer screening among women who were either underscreened or had never been screened [32].

The advertisement campaign will be launched at the same time across all study arms for a period of 4 weeks, based on the suggested campaign timeframe from the public relations firm following their media sufficiency exercise [33,34]. We will set a maximum budget for the campaign and split the funds equally among the 5 study arms that will be shown FB advertisements. Since the population of rural FSAs is lower, we will allocate 85% of the budget for each arm to be spent on urban FSAs and 15% to be spent on rural FSAs. This will ensure that the advertisements will be shown to both rural and urban FB users and the budget is proportional to the population of urban and rural FSAs in each arm.

### Randomization and Allocation

Stratified randomization using computer-generated random numbers will be used to allocate FSAs to one of the 6 study arms (Figure 1). In 4 arms, eligible FB users will all receive one of the 4 social media advertisements developed in previous studies [29]. In the fifth arm, we will test a tailored strategy by sex. The advertisements selected for the tailored strategy were identified from our 2 split (A and B) tests conducted to help us select advertisements for the RCT. Briefly, the top-performing advertisement with female users in the first split test and the top-performing advertisement with male users in the second split test were selected for the tailored strategy. These advertisements are presented in Multimedia Appendix 1. These 5 arms will be used for the current analysis of screening intention and user engagement. In the sixth and final arm, FB users will not be shown any advertisements. This arm will serve as a control group for future work that will explore the effectiveness of the advertisements on actual screening uptake by linking health administrative data to explore screening participation in each of the trial arms in comparison to the control arm. Randomization will be performed by a clinical epidemiologist external to the study team who has not been involved in study design and will not participate in the conduct of the study or analysis of the results. The study team will therefore be blinded regarding which FSAs are allocated to which trial arm. Randomization will be stratified based on the rural-urban nature of the FSA to ensure that we have an approximately equal assignment of rural FSAs to each trial arm.

### Outcome Measures

#### Primary Outcome

The primary outcome for analysis is the number of people per FSA who pledge their intention to get screened for CRC. Intention is an important precursor to action [35], and there is a strong association between intention and screening participation [36-39]. Intention is therefore commonly used as a surrogate end point in cancer screening trials [40-44]. The goal is to identify which advertisement performs best in relation to one another and the tailored strategy.

Wide variability exists in how screening intention has been captured in prior work, with no evidence of a gold-standard or validated tool [41,45-49]. Prior studies have used a range of Likert-type scales to measure screening intention or yes or no questions that ask about intention to get screened within a specified timeframe. For example, Christou and Thompson [46] measured CRC screening intention by asking participants if they would consider doing a fecal occult blood test bowel cancer screening test in the next 6 to 12 months if a screening kit was given or sent to them. Others have measured it by asking “Imagine you have just turned 60 and have received a bowel screening test kit (FOBT test kit) in the post, would you do the test?” with response options on a 4-point Likert scale, including “yes, definitely,” “yes, probably,” “probably not,” and “definitely not” [48]. Similarly, Schroy et al [49] used a 5-point Likert scale ranging from 1 for “very unsure” to 5 for “very sure” to measure how sure participants were that they would schedule a CRC screening test and how sure participants were that they would complete the screening test scheduled. In their prior study, OH measured screening intention through the concept of “pledging” where users were able to view an intentions drop-down list (Figure 2) and select their intentions, if any, regarding breast cancer screening. We will use this approach, adapted for CRC screening, to measure screening intention given the lack of a validated tool that has been consistently used in previous research. The pledge is delivered on the website where users are redirected if they click on any of the advertisements. We will use Meta Pixel to capture the website visitors’ actions. We will be able to track how many FB users click on the advertisements, visit the website, complete the pledge form, and from which trial arm website visitors and/or pledgers originate from. Google Analytics from the website itself will also capture the time of day a user visited the site, the type of device they were using, and whether they were a new or returning user.

#### Secondary Outcomes

Secondary outcome measures (Textbox 1) will focus on engagement metrics for each of the 5 arms where advertisements will be shown as captured through FB Ads Manager, the interface that allows users to set up, manage, and track the performance of their advertisements on FB.

**Secondary outcomes of user engagement.**

**Cost per click**

- Average cost per link click calculated as the total amount spent on the advertisement divided by the total number of clicks

**Reach**

- Number of people who viewed the advertisement at least once

**Click-through rates**

- Percentage of people who click on an advertisement after seeing it

**Number of likes**

- Total number of Facebook likes per advertisement

**Number of impressions**

- Number of times that each advertisement was on screen; may appear on a user’s screen more than once, differentiating impressions from Reach

**Post comments**

- Number of comments on each ad

### Statistical Analysis

Baseline descriptive characteristics will be reported for each study arm, including the total number of urban and rural FSAs randomly allocated to each arm and the number of eligible FB users (including the proportion of men and women). For the primary outcome (screening intention), any response checked on the pledge menu that indicates the user intends to take action toward screening (answers 1-3) will be grouped and recoded as “has intention to screen” [50].

Primary analysis—the number of people per FSA who pledge an intention to screen will be analyzed using an FSA-aggregated count model, with Poisson regression as the primary approach and a negative binomial model used if overdispersion is present. Models will include an offset for the number of eligible FB users reached in each FSA (to estimate rate ratios) and will account for the stratification factor (urban or rural) as appropriate. Estimate rate ratios with 95% CIs will be reported. Sensitivity analyses will assess robustness to alternative modeling choices (eg, quasi-Poisson or additional adjustment for baseline FSA-level characteristics if available).

Secondary analyses—engagement metrics (cost per click, reach, click-through rate, number of impressions, and post comments) will be summarized by trial arm and by urban or rural FSA; for the tailored strategy, summaries will also be stratified by sex. These outcomes will be analyzed descriptively and, where formal comparisons are undertaken, appropriate regression models for counts or rates will be used with FSA-level inference.

### Sample Size and Power

In total, 521 FSAs will be randomized into 6 arms (approximately 86-87 FSAs per arm). The primary analysis focuses on the 5 arms that receive FB advertising. Because the number of FSAs is fixed, we estimated the smallest detectable difference in the primary outcome (number of pledges per FSA) under 80% power and a 2-tailed α of .005 (Bonferroni corrected for 10 pairwise comparisons). We used the approach of Gu et al [50]:



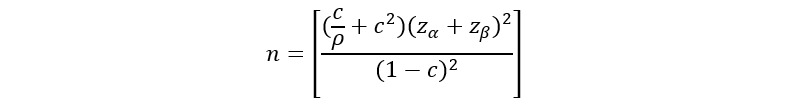



where ρ=1, *c* is the ratio of the 2 rates being compared z_α_=2.576, z_β_=0.842. Using n=86 FSAs per arm yields a detectable rate ratio of c=0.63. Thus, with 86 FSAs per arm, we have 80% power to detect relative differences of approximately 37% or greater between arms.

We anticipate some heterogeneity across FSAs in population size and engagement; however, randomization and stratification by urban or rural status should balance these factors across study arms. In the primary analysis, pledge counts will be modeled as rates using the number of eligible FB users reached in each FSA as the denominator, and models allowing for overdispersion (eg, negative binomial or quasi-Poisson) will be used if extra-Poisson variation is present.

### Future Analysis

A future analysis is planned to evaluate actual screening uptake at the FSA level using linkage to health care administrative data at ICES (formerly known as the Institute for Clinical Evaluative Sciences). For this analysis, all 6 trial arms will be used. CRC screening rates will be compared between residents of FSAs receiving social media advertisements and those not receiving them, over 6 months postmarketing campaign.

## Results

Anticipated results of this study include the number of people per FSA who pledge their intention to screen for CRC as well as engagement with the advertisement campaign (number of link clicks, cost per click, click-through rate, number of impressions, and post comments for each trial arm). Our main outcome of interest, screening intention, will be captured through a pledge form available on the website where those who click on the advertisement will be redirected. We will be able to compare screening intention across trial arms through the comparison of pledges completed by users allocated to one of the 5 trial arms. We will also conduct a sensitivity analysis to explore whether there are any differences in screening intention between rural and urban FSAs. Similarly, we will report on the engagement metrics for each of the trial arms and compare whether engagement was higher in one trial arm than in another. We will also compare the results by urban and rural FSAs to explore whether engagement differed among users in these clusters. This information may provide insights into what types of messages FB users of 45 to 64 years are most drawn to. Status of data analysis and expected results to be published in May 2027.

## Discussion

This study has the potential to inform CRC screening programs at local, national, and international levels. Our study will assess whether social media can effectively deliver CRC screening messages and determine which messages best encourage screening intentions. Our approach is also innovative as it could help contain intervention costs by setting a maximum budget to be used for the social media campaign across all study arms. This will be of particular interest to other researchers and organizations with a prespecified budget and will have the potential to reach many people in a relatively short amount of time. Given the increasing internet access and daily use of social media platforms by individuals of screen-eligible age, our research is needed to better understand how social media can be leveraged for the promotion of cancer screening—an approach that has not been widely explored [22]. Additionally, our intervention may be of interest to jurisdictions tasked with prevention and cancer screening efforts as it is a low–resource-intensive intervention compared with other strategies (eg, patient navigation [7]). Our intervention can be organized from anywhere with internet access and can be targeted and delivered to individuals around the world with the capacity to target users based on interests, age, sex, location, behavior, and other demographics.

The results of our study will need to be considered in light of the study limitations. We are unable to solely target individuals of screen-eligible age in our advertisement campaign due to the limitations of the social media platform itself. As such, we chose to target those aged 45 to 64 years for this trial, the category that most closely matches CRC screening age in Canada. Recent data show that the incidence of CRC is increasing among those even younger than 50 years of age [51], and in some jurisdictions, screening for those aged 45 years is recommended [52,53]. Moreover, there is an active discussion in Canada about the potential to lower the CRC screening age to 45 [54,55]. We will not collect information on individual targeted FB users and thus will be unable to determine outcomes at the individual level. We also recognize that FB’s algorithmic optimization may introduce bias through the potential differential exposure intensity across the trial arms. To address the implications of this, we will turn off automatic, algorithmic budget reallocation and instead split the funds equally among the 5 trial arms. We will also ensure proportional allocation of the budget across urban and rural FSAs to ensure that the advertisements are shown to users in both urban and rural locations.

Despite these limitations, our study will provide important insights about the use of social media for the promotion of CRC screening with engagement metrics for each of the trial arms, providing information about what types of messaging may encourage higher user engagement and screening intention.

Our study results may be translated into practice at OH, especially given our strong collaboration with the organization. Additionally, our results may be leveraged by other screening programs looking for new ways to promote CRC screening. Our results are likely to be considered during various campaigns for CRC awareness month (March), one specific example of how organizations could leverage our findings. Our study results and methodology specifically will be easily translatable to other cancer disease sites with screening programs, including breast and cervical cancer screening, or to other social media platforms.

## Appendix

Multimedia Appendix 1 Social media advertisements to be tested in the trial arms.

## Abbreviations

CCC: ColonCancerCheck
CONSORT: Consolidated Standards of Reporting Trials
CRC: colorectal cancer
FB: Facebook
FSA: forward sortation area
OH: Ontario Health
RCT: randomized controlled trial
SPIRIT: Standard Protocol Items: Recommendations for Interventional Trials
