# Supplementary material for: Using Facebook to Improve Participation in Colorectal Cancer Screening: Protocol for a Cluster Randomized Controlled Trial
Source: JMIR Res Protoc. 2026 May 14;15:e86829. doi: 10.2196/86829 (PMC13219987; doi:10.2196/86829)
Supplement: Multimedia Appendix 1 [file resprot_v15i1e86829_app1.docx]

**Multimedia Appendix 1.** Social media advertisements to be tested in the trial arms.

| Trial Arm 1: Social media ad #1 | |
| --- | --- |
| 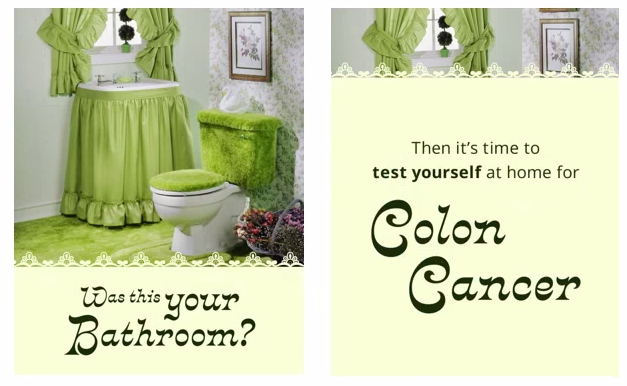 | **Ad Text**  Remember this bathroom? Adults aged 50+ are at increased risk of colon cancer.  Getting checked for colon cancer is easy and can be done in the privacy of your own home.  Learn more button: https://www.getcheckedtoday.ca/ |
| Trial Arm 2: Social media ad #2 | |
| 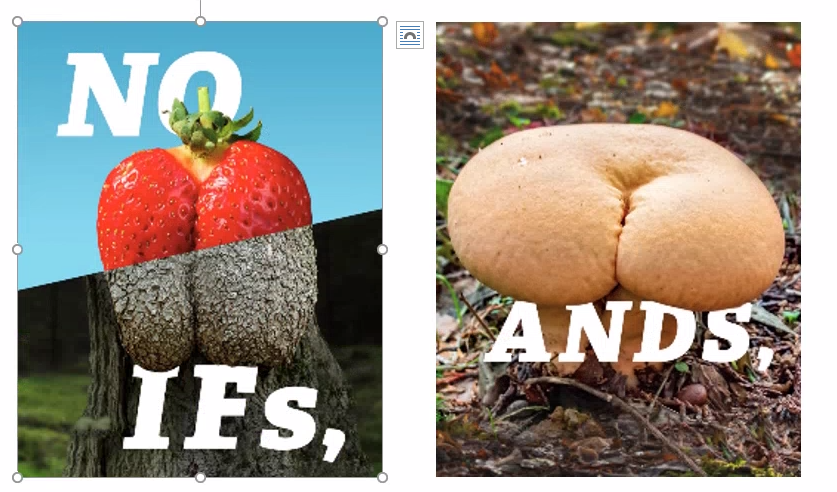 | **Ad Text**  No ifs, ands, just butts.  Test yourself for colon cancer today.  It’s easy and can be done at home.  Learn more button: https://www.getcheckedtoday.ca/ |
| Trial Arm 3: social media ad #3 | |
| 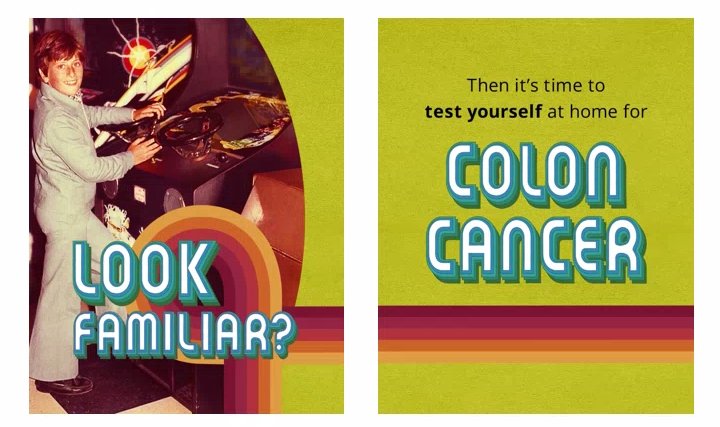 | **Ad Text**  Look familiar? Adults aged 50+ are at increased risk of colon cancer.  Health care providers recommend you get checked for colon cancer every 2 y.  Learn more button: https://www.getcheckedtoday.ca/ |
| Trial Arm 4: social media ad #4 | |
| 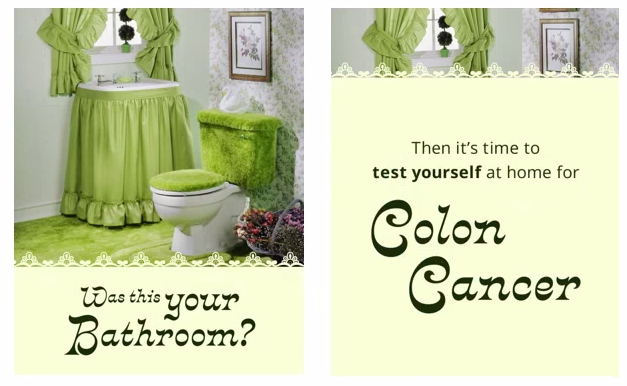 | **Ad Text**  Do not flush it away. Adults aged 50+ are at increased risk of colon cancer.  Test yourself for colon cancer today. It’s easy and can be done at home.  Learn more button: https://www.getcheckedtoday.ca/ |
| Trial Arm 5: tailored Strategy (Show the best performing ad to men and best performing ad to women.) | |
| Women: | |
| 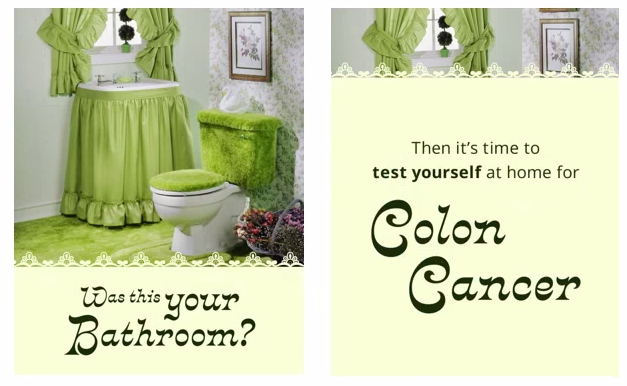 | **Ad Text**  Remember this bathroom? Adults aged 50+ are at increased risk of colon cancer.  Getting checked for colon cancer is easy and can be done in the privacy of your own home.  Learn more button: https://www.getcheckedtoday.ca/ |
| Men: | |
| 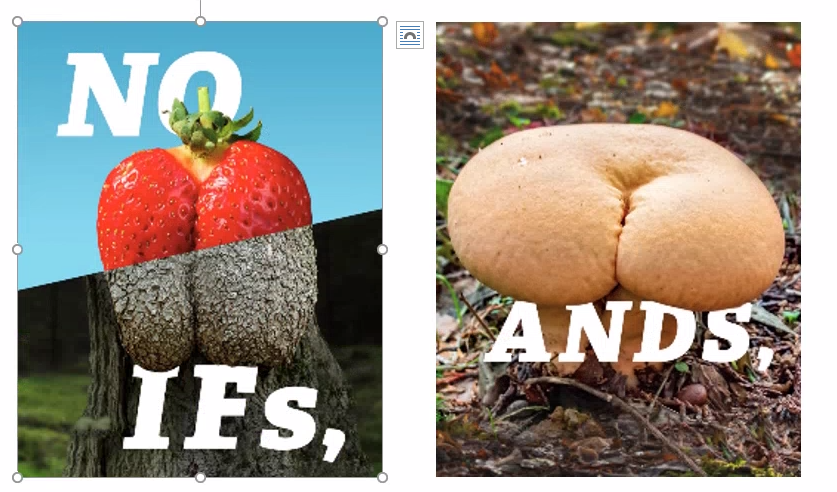 | **Ad Text**  No ifs, ands, just butts.  Test yourself for colon cancer today. It’s easy and can be done at home.  Learn more button: https://www.getcheckedtoday.ca/ |
| Trial Arm 6: No message (control/comparison group) | |
